# Adaptation and validation of the Evidence-based Practice Profile Questionnaire (EBP^2^Q) for clinical postgraduates in a Chinese context

**DOI:** 10.1186/s12909-023-04594-6

**Published:** 2023-08-21

**Authors:** Yitong Jia, Xinqi Zhuang, Yao Zhang, Ge Meng, Shijia Qin, Wen-Xin Shi, Xiaojian Wu, Yin-Ping Zhang

**Affiliations:** 1 Faculty of Nursing, Xi’an Jiaotong University Health Science Center, No.76, West Yanta Road, Xi’an, Shaanxi 710061 P.R. China; 2 School of Nursing, Shenzhen University Health Science Center, Shenzhen, Guangdong 518060 China; 3 Department of Graduate Education, Xi’an Jiaotong University Health Science Center, Xi’an, Shaanxi 710061 China

**Keywords:** Evidence-based practice, Clinical postgraduates, Professional degree postgraduates, Reliability, Validity

## Abstract

**Background:**

Evidence-based practice (EBP) is an essential approach of optimizing patient outcomes and driving progress in clinical practice. As an important reserve talent of medical staff and researchers, the clinical postgraduates are expected to become the backbones of supporting the implementation of EBP in clinical units after graduation. The assessment of their EBP learning outcomes is an important issue, yet few tools have been developed specifically in Mainland China. The purpose of this study is to adapt the Evidence-Based Practice Profile Questionnaire (EBP^2^Q) to Mainland China’s cultural context and to evaluate the psychometric properties of the Chinese EBP^2^Q in clinical postgraduates.

**Methods:**

Cross-cultural modification, including translating the original EBP^2^Q into Chinese was implemented according to established guidelines. A pilot study was carried out in Mainland China among 30 clinical postgraduates. A subsequent validation study was conducted among 633 clinical postgraduates majoring in clinical medicine, stomatology and nursing from Mainland China. Construct validity was assessed by exploratory factor analysis (*n* = 313), together with confirmatory factor analysis (*n* = 320). Reliability was determined by internal consistency and test-retest reliability.

**Results:**

The Chinese EBP^2^Q consisted of 40 items. The content validity index of the Chinese EBP^2^Q achieved 0.938 at an acceptable level. Principal component analysis resulted in a four-factor structure explaining 61.586% of the total variance. All fitting indices satisfied the standard based upon confirmatory factor analyses, indicating that the four-factor structure contributed to an ideal model fit. The internal consistency appeared high for the Chinese EBP^2^Q, reaching a Cronbach’s alpha value of 0.926. Test–retest reliability was 0.868 and the split-half coefficient was 0.925.

**Conclusion:**

Chinese version of EBP^2^Q possesses adequate validity, test-retest reliability and internal consistency. It is a promising questionnaire to be adopted by Chinese medical educators in designing their course and curriculum, or by clinical postgraduates for self-assessment of EBP learning.

## Background

With the quickened pace of industrialization and urbanization, as well as its remarkable impact on the improvement of people’s living standards, public demands for healthcare services have increased accordingly. The medical model has transformed from simple disease treatment to a combined mode of prevention, care, treatment and rehabilitation. In China, it has been proposed that the development goals of healthcare undertakings include providing people with a full range of full-cycle healthcare services [1]. In such a circumstance, it has become an urgent need to strengthen the construction of compound medical talent team, accelerate the innovative development of medical education and cultivate more high-level and application-oriented talents for medicine and healthcare.

To meet the requirements of the national medical education strategy, Chinese government has been committed to promoting the reform of the postgraduate education in the past decade, and has taken the development of professional degree postgraduate education as a significant national policy. The professional degree postgraduate education is a new form of postgraduate education in China. Compared with the academic degree postgraduate, it focuses more on cultivating advanced applied talents and providing practiced manpower [2]. As an important reserve of medical staff and researchers, clinical professional degree postgraduates are expected to complete various work and research in clinical units in the future. They will play a key role in improving the overall level of health service and achieving the goal of national health [3], which requires them to possess both profound professional knowledge and excellent clinical manipulation skills, as well as critical thinking and the ability of implementing Evidence-based practice (EBP) [4, 5].

EBP has gained increasing popularity worldwide. It requires health professionals to use the best available evidence when making medical decisions. In addition to the contexts and preferences of individual patients, the evidence provided by authoritative research results will contribute to generating best practice behaviors and optimizing patient outcomes [6, 7]. It has become a norm that health professionals are supposed to demonstrate evidence-based practice behaviors on a daily basis [8, 9]. The clinical postgraduates, especially professional degree postgraduates, will become backbones to support EBP in clinical units after graduation [3]. It is useful for them to have sufficient knowledge and skills in conducting literature search and critical appraisal of evidence [10]. Thus, medical educators should identify the effectiveness of EBP programs and determine the best method to teach students the knowledge and skills required for EBP. Currently, many medical schools around the world have tried to incorporate evidence-based medicine teaching programs into their curriculum system [11]. A crucial aspect in evaluating education programs is to choose instrument for evaluating the effect of educational teaching [12]. Selecting an instrument to best assess the effectiveness of EBP learning outcomes is necessary.

Several instruments existing for evaluating EBP are relevant to medical students in foreign countries, including Fresno Test [13], Berlin Questionnaire [14], Assessing Competency in Evidence Based Medicine (ACE) Tool [15], Evidence-Based Practice -Knowledge, Attitude and Behavior (KAB) Questionnaire [16] and Evidence-Based Practice Profile Questionnaire (EBP^2^Q) [17]. Some instruments were cross-culturally adapted to measure EBP for nursing students or nurses in Mainland China [18, 19]. However, none of them was developed specifically for clinical postgraduates. Existing domestic instruments for evaluating EBP of clinical postgraduates were mostly self-designed to test their attitudes, behaviors or beliefs towards EBP, which had shortcomings in contents and psychometric properties. The EBP^2^Q was developed at the University of South Australia by Maureen McEvoy et al., and was validated on 526 people (consisting of 481 students and 45 academics/practitioners). Apart from its good psychometric parameters, an additional advantage of the EBP^2^Q is that it can be applied to self-assessment of the knowledge, skills, attitudes and behaviors required for EBP by students, lecturers, and practitioners. It can also be applied to assess different aspects of EBP by selecting individual parts (domains) of the questionnaire [17]. Therefore, we cross-culturally adapted the EBP^2^Q to measure EBP learning outcomes of Chinese clinical postgraduates and subsequently evaluated its psychometric properties.

## Methods

### Participant and setting

A cross-sectional validation study was conducted with 633 clinical postgraduates (major in clinical medicine, stomatology and nursing) using convenience sampling from three university affiliated hospitals (the First Affliated Hospiatl of Xi’an Jiaotong University, the Second Affiliated Hospital of Xi’an Jiaotong University and Hospital of Stomatology Xi’an Jiaotong University) in northwest China. Participant inclusion criteria included: (a) enrolled in a Master or Doctor of Clinical postgraduate degree program; (b) possessing 3 months or more in clinical practice and already adjusting themselves to the working environment; (c) willing to participate in the study. All participants were informed consent after the study aims and procedures had been fully explained. Anonymity and confidentiality were assured and participants were told that they could withdraw at any point without consequences. Data were collected by the online survey utilizing sojump (an online research survey tool; http://www.sojump.com). Approval was obtained from the Ethics Committees of Xi’an Jiaotong University. All procedures followed were in accordance with the ethical standards of the Declaration of Helsinki.

### Instruments

#### The Evidence-based Practice Profile Questionnaire (EBP^2^Q)

The original Evidence-based Practice Profile Questionnaire (EBP^2^Q) was developed in 2010 in Australia, which was initially developed with academics and students from health and non-health backgrounds to assess knowledge and skills in EBP. The original instrument comprised five distinct domains: Relevance, Sympathy, Practice, Terminology and Confidence. Relevance (14 items) refers to the value, emphasis and importance placed on EBP; Sympathy (7 items) refers to the individual’s perception about the compatibility of EBP with professional work; Terminology (17 items) refers to the understanding of common research terms; Practice (9 items) refers to the use of EBP in clinical situations and Confidence (11 items) refers to the perception of an individual’s ability with EBP skills. All items are scored on a 5-point Likert scale and the items in Sympathy domain are reversely scored. The 58-item questionnaire demonstrated acceptable internal consistency (Cronbach’s alpha 0.96) and test–retest reliability. When compared to the instrument developed by Upton & Upton [20], the EBP^2^Q was shown to have good convergent validity in the three comparable factors (Practice 0.66, Confidence 0.80 and Sympathy 0.54). Descriptive statistics and correlation coefficients demonstrated sufficient item facility and discrimination of the original EBP^2^Q. As a well-developed instrument, the original EBP^2^Q version was examined strong psychometric properties.

#### General Information Questionnaire

Socio-demographic and evidence-based practice relevant data were obtained through the General Information Questionnaire that we developed. The questionnaire includes age, gender, specialty, educational background, degree (academic degree or professional degree), English level, clinical practice duration, clinical work experience, research experience on EBP, supervisor’s research experience on EBP, interests in EBP, EBP courses or training duration, and necessary to implement EBP courses or training.

### Translation procedure

After obtaining original author approval, translation and cross-cultural adaptation of the EBP^2^Q were performed according to a clear and user-friendly guideline [21]. The guideline outlines a thorough adaptation process of self-report measures, aiming to maximize the attainment of semantic, idiomatic, experiential, and conceptual equivalence between the source and target questionnaires. The adaptation process can be carried out within the following stages as recommended: translation (Stage I), synthesis (Stage II), back translation (Stage III), expert committee review (Stage IV), pretesting (Stage V), together with further testing of the adapted version and evaluation of the adaptation process (Stage VI). The EBP^2^Q was adapted to Chinese in strict adherence to the guideline.

The original English version was independently translated into Chinese by three translators who respectively work in clinical department, evidence-based medicine education department and English language teaching department. The differences between three completed translation versions were then resolved after comprehensive discussion with the participation of the fourth translator, and they ultimately accomplished a forward-translated version of questionnaire. Subsequently, the questionnaire was back translated independently by another two independent translators (i.e., bilingual experts fluent in English and Chinese) who were blind to the original English version. A multidisciplinary consensus committee comprised by one methodologist (a member of the research team), one health care professional, five bilingual and bicultural translators was held to consolidate all the translated and back translated versions of the questionnaire, verify any controversial or ambiguous wording, ensure cross-cultural equivalence and develop the pre-final version of the questionnaire for field testing.

### The pilot study

Chinese version of the questionnaire was tested on a sample of 30 postgraduates majoring in clinical medicine, stomatology and nursing, which were recruited through convenience sampling in Northwest China. All volunteers were asked to complete the questionnaires. The pilot study enabled us to detect problems with wording, terminology, instructions and the clarity of options. An interview was conducted to explore their perception and understanding of each item, and to take their advice for the improvement of the questionnaire. The interview was uniformly performed by the researcher to address three aspects: the instruction of the questionnaire, the content of the entries, and the domain of the entries. The outline of the interview was as follows: Q1: Do you have any suggestions for the instruction of the questionnaire? Q2: Do you have any suggestions for the domains of the entries? Q3: Which entries do you find difficult to comprehend? Q4: Do you have any recommendations for the wording of the entries? This process ensured that the adapted versions were still retaining the equivalence and linguistically appropriate when applied in practice. After this process, the final Chinese version was developed [18, 22]. The 30 students were recruited to complete the same questionnaire to measure the test-retest reliability 2 weeks later.

### Data analysis

The Statistical Package for the Social Sciences (SPSS) version 19.0 was used for data analysis. The statistical description of the demographic variable was carried out by frequency tables, the means, and the standard deviations (SD). The 7 experts were invited to judge the degree of relevance of each item based on the recommended 4-point scale from 1 (very invalid) to 4 (very valid) for the content validation. Content validity index (CVI) was computed to quantify scores for each item and the whole questionnaire. Items rated at a 3 or 4 on the 4-point relevance scale suggest expert have reached consensus regarding relevance. The content validity index of each item (I-CVI) and the overall scale (S-CVI) were calculated, and an S-CVI of more than 0.90, together with I-CVIs of more than 0.78 were denoted validity [23]. The internal consistency was calculated with Cronbach alpha coefficient. The Cronbach’s alpha value of 0.7 or greater was considered satisfactory [24]. Split-half coefficient reliability was assessed by using half of odd and even items. Test-retest reliability was assessed by using the intraclass correlation coefficient (ICC) for the whole questionnaire and each domain [25]. ICC values of 0.60 to 0.80 were deemed good reliability, and ICC values above 0.80 were regarded as excellent reliability [26]. Validity of each item was determined through item analysis. We considered unfavorable floor or ceiling effects to be present if more than 15% of the individuals reached the highest or lowest score. Exploratory factor analysis using Principal component analysis (PCA) as the extraction method and Direct Oblimin as the rotation method was conducted to determine the factor structure of questionnaire [27]. The number of factors was identified with the following strategies: (a) The Kaiser Criterion (eigenvalue), (b) the “elbow” joint in the scree plot, (c) the clinical interpretability. Items were deemed relevant if factor-loading coefficients exceeded 0.40 and extracted factors achieved an eigenvalue ≥ 1.0 [28]. A confirmatory factor analysis (CFA) was also performed to verify the results. The expected values of indices recommended were as follows [29]: (a) Chi-squared divided by the degrees of freedom ≤ 3; (b) the root mean squared error of approximation (RMSEA) < 0.08; (c) the comparative fit index (CFI), normed fit index (NFI) and goodness-of-fit index (GFI) > 0.90.

## Results

### Translation and adaptation of EBP^2^Q

The forward and backward translations were repeated three times until an acceptable version was obtained. According to expert enquiry, the Likert-5 scoring options set after items 1 to 8 in the Relevance domain were uniformly revised as “5 = strongly agree”, “4 = agree”, “3 = neutral”, “2 = disagree” and “1 = strongly disagree”. The supplementary interpretation was given in item 22 “Formulated a clearly answerable question that defines the client or problem, the intervention and outcome(s) of interest” to ensure that respondents could understand, that was “construct clinical questions using the principles of PICO”. The words “set standards” in item 55 were modified into “existing standards for reference evaluation” as needed for cross-cultural adaptation.

### Pilot study

The pre-final Chinese EBP^2^Q was tested on a sample of 30 postgraduates majoring in clinical medicine (40%), stomatology (30%) and nursing (30%), which were recruited through convenience sampling in Northwest China. In order to better fit Chinese context, the phrase “develop knowledge” in item 5 was suggested to be translated as “expand knowledge”, and the term “client” in the whole questionnaire was recommended to be translated as “service recipients”. One of the participants proposed to revise item 28 “Read published research reports” into “Read published research reports related to EBP”, as the scope of “research reports” was too broad and not specific enough to reflect the Practice domain of EBP. The researcher recorded participants’ suggestions during the pilot study and made modifications after discussing with the multidisciplinary consensus committee.

### Validation study

#### Sample characteristics

Demographic data of the validation study are presented in Table 1. The total number of participants included 633 clinical postgraduates (postgraduates of clinical medicine accounted for 72.8%, stomatology accounted for 13.6%, and nursing accounted for 13.6%). The mean age was 25.18 years (SD = 2.24). Participants included 465 (73.5%) female and 168 (26.5%) male who were in their Master (80.7%) and Doctor (19.3%) of postgraduate degree programs. Over half (56.7%) received EBP courses or training and 52.3% of participants were very interested in EBP. 49.4% of the participants had research experience, while almost half of them (52.1%) had research experience on EBP. A total of 608 participants (96.1%) strongly agreed that it was necessary to implement EBP in clinical settings.

**Table 1:** Characteristics and EBP related information of sample (*n* = 633)

Item	n (%)
Gender	
Female	465 (73.5)
Male	168 (26.5)
Specialty	
Clinical medicine	461 (72.8)
Stomatology	86 (13.6)
Nursing	86 (13.6)
Education background	
Master	511 (80.7)
Doctor	122 (19.3)
Degree	
Academic degree	163 (25.8)
Professional degree	470 (74.2)
Clinical practice duration	
3–12 months	375 (59.2)
13–24 months	119 (18.8)
25–36 months	139 (22.0)
Clinical work experience	
Yes	417 (65.9)
No	216 (34.1)
Research experience	
Yes	313 (49.4)
No	320 (50.6)
Research experience on EBP (*n* = 313)	
Yes	163 (52.1)
No	150 (47.9)
Supervisor’s research experience on EBP	
Yes	266 (42.0)
No	107 (16.9)
Unclear	260 (41.1)
English level	
CET 6 below	75 (11.8)
CET 6 & CET 6 above	558 (88.2)
Interests in EBP	
Very interested	331 (52.3)
A little interested	302 (47.7)
Received EBP courses or training	
Yes	359 (56.7)
No	274 (43.3)
EBP courses or training duration (*n* = 359)	
1–12 h	116 (32.3)
13–24 h	131 (36.5)
More than 24 h	112 (31.2)
Necessary to implement EBP courses or training	
Yes	608 (96.1)
No	25 (3.9)

College English Test Band 6 (CET-6) is a benchmark to test the college students’ English proficiency in China

#### Item analysis

First, we sorted EBP^2^Q items into high and low scoring groups according to the total score of participants. The top 27% of the highest scoring items comprised the high group, and the lower 27% of the lowest scoring items comprised the low group. Then the mean score of each item in the two groups was compared using independent samples t-test to test the difference between two groups, and the critical ratio (CR) of the item was obtained. The results showed that there was a statistically significant difference in the scores of each item between the high group and the low group (*p* < 0.001), and the CR value of each item was greater than 3, indicated that every item had good discrimination without the floor or ceiling effect. No entries were deleted at this stage.

#### Content validity

The CVI evaluation form was distributed to the seven experts who were asked to rate content validity. All experts agreed that the EBP^2^Q was particularly designed to measure the EBP learning outcomes. S-CVI of the questionnaire reached 0.938, which indicated excellent content validity. I-CVIs were above 0.78 except items 1–4 in the Relevance domain and items 31, 32, 34, 36, 40, 42, 43 in the Terminology domain. These 11 items were deleted because of a low validity. In addition, the experts suggested merging or deleting some items in the Sympathy domain and the Relevance domain for they expressed the similar meaning. For example, items 15, 16, 20 and 21 in the Sympathy domain were basically the same as the items 9, 10, 11 and 14 in the Relevance domain, respectively. The major difference between them was the scoring method, while the items in the Sympathy domain were negatively worded and required to be reverse scored. According to the experts, the other items in the Sympathy domain (items 17, 18 and 19) involved the accumulation of long-term clinical work experience of the respondents, while our questionnaire was designed for medical students with limited clinical work experience. Therefore, we deleted the items of the Sympathy domain (items 15–21) after discussion. The final version of Chinese EBP^2^Q consists of 40 entries. The results are shown in Table 2.

**Table 2:** The content validity of each item of the Chinese EBP^2^Q

Item	I-CVI	Item	I-CVI	Item	I-CVI
^a^R1	0.714	P22	1.000	^a^T43	0.714
^a^R2	0.714	P23	1.000	T44	1.000
^a^R3	0.714	P24	1.000	T45	1.000
^a^R4	0.714	P25	1.000	T46	1.000
R5	1.000	P26	1.000	T47	0.857
R6	1.000	P27	1.000	C48	1.000
R7	1.000	P28	1.000	C49	1.000
R8	1.000	P29	1.000	C50	1.000
R9	1.000	P30	1.000	C51	1.000
R10	1.000	^a^T31	0.714	C52	1.000
R11	1.000	^a^T32	0.714	C53	1.000
R12	1.000	T33	1.000	C54	1.000
R13	1.000	^a^T34	0.714	C55	1.000
R14	1.000	T35	1.000	C56	1.000
^a^S15	1.000	^a^T36	0.714	C57	1.000
^a^S16	1.000	T37	1.000	C58	1.000
^a^S17	1.000	T38	0.857		
^a^S18	1.000	T39	0.857		
^a^S19	1.000	^a^T40	0.714		
^a^S20	1.000	T41	1.000		
^a^S21	1.000	^a^T42	0.714		

R, S, P, T & C represent the domains of Relevance, Sympathy, Practice, Terminology, and Confidence in the original English version of EBP^2^Q respectively

^a^Indicates the entry needs to be deleted

#### Exploratory factor analysis

**Fig. 1:**
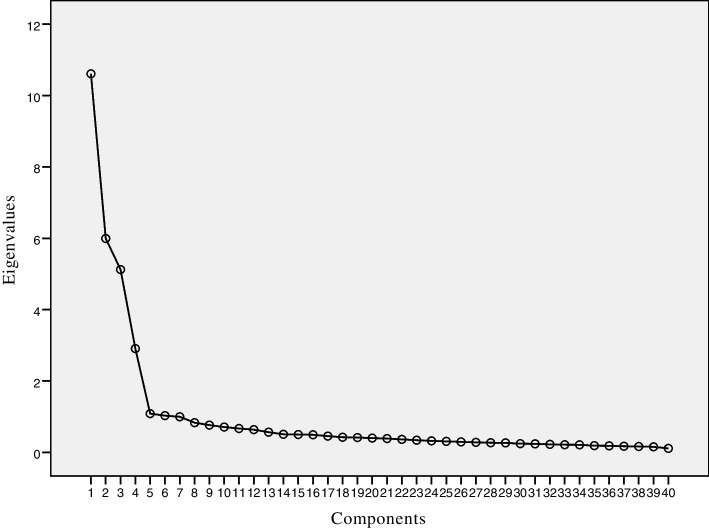
Scree plot of the Chinese version of EBP^2^Q (*n* = 313)

The data was divided into two parts randomly in this study. The first 313 samples were employed for exploratory factor analysis using oblique rotation to account for the relationship among the factors. The correlation matrix showed ample adequacy of the sample size (the Kaiser-Meyer-Olkin measure was 0.922) and the Bartlett test results (χ^2^ = 13,882.106, *p* < 0.001) rejected the hypothesis of zero correlations. The scree plot (Fig. 1) indicated that there were four factors. In addition, based on Kaiser’s criterion of extracting factors with eigenvalues of greater than 1, a four-factor structure (Factor 1 = 10.610, Factor 2 = 5.994, Factor 3 = 5.124, Factor 4 = 2.907) that explained 61.586% of the variance of the data was identified by the pattern matrix (see Table 3). Exploratory factor analysis of the 40 items produced factor loading from 0.565 to 0.872. Factor 1 comprised 11 items (items 48–58), all taken from the Confidence domain; Factor 2 comprised 10 items (items 33, 35, 37–39, 41, 44–47), all taken from the Terminology domain; Factor 3 comprised 10 items (items 5–14), all taken from the Relevance domain; Factor 4 comprised 9 items (items 22–30), all taken from the Practice domain. Combined with the results of scree plot, Kaiser Criterion (eigenvalue) and the meaningfulness of factors [30], a four-factor structure was identified. Correlation analysis showed a weak correlation between the extracted factors (factor intercorrelations ranged from 0.137 to 0.461), indicating the suitability of the oblique solution [31].

**Table 3:** Factor loadings on items of the EBP^2^Q (*n* = 313)

Item No	Domain	Item	Factor 1	Factor 2	Factor 3	Factor 4	Communalities
52	C	Awareness of major information types and sources	**0.872**	0.170	0.128	0.376	0.764
55	C	Ability to critically analyses evidence against existing standards for reference evaluation ie quality scoring	**0.855**	0.110	0.112	0.387	0.739
54	C	Ability to access evidence (get copies of articles or reports)	**0.845**	0.208	0.093	0.392	0.747
51	C	Ability to convert your information needs into clearly answerable questions	**0.845**	0.129	0.107	0.400	0.720
56	C	Ability to determine how valid (close to the truth) the material is	**0.845**	0.095	0.139	0.435	0.724
58	C	Ability to apply information to individual cases	**0.844**	0.070	0.151	0.415	0.720
57	C	Ability to determine how useful (clinically applicable) the material is	**0.830**	0.118	0.153	0.414	0.693
53	C	Ability to search an electronic database	**0.790**	0.200	0.079	0.340	0.737
50	C	Ability to identify gaps in your knowledge	**0.784**	0.125	0.176	0.344	0.622
48	C	Research skills	**0.772**	0.074	0.126	0.398	0.606
49	C	Computer skills	**0.766**	0.155	0.094	0.349	0.592
44	T	Randomized controlled trial (RCT)	0.047	**0.849**	0.149	0.024	0.757
46	T	Continuous outcomes	0.083	**0.829**	0.163	0.028	0.711
45	T	Dichotomous outcomes	0.093	**0.820**	0.103	0.054	0.676
35	T	Meta analysis	0.121	**0.815**	0.109	0.164	0.683
37	T	Confidence interval	0.115	**0.803**	0.111	0.006	0.661
38	T	Publication bias	0.152	**0.794**	0.094	0.071	0.653
41	T	Statistical significance	0.058	**0.780**	0.150	0.080	0.683
39	T	Forest plot	0.210	**0.729**	0.033	0.178	0.636
33	T	Systematic review	0.176	**0.724**	0.153	0.162	0.595
47	T	Treatment effect size	0.235	**0.662**	0.103	0.203	0.563
11	R	I need to increase the use of evidence in my daily work	0.122	0.134	**0.833**	0.134	0.734
12	R	I am interested in learning or improving the skills necessary to incorporate EBP into my work	0.144	0.129	**0.799**	0.144	0.692
9	R	Application of EBP is necessary in my work	0.083	0.108	**0.793**	0.115	0.636
7	R	I intend to read relevant literature to update knowledge	0.064	0.154	**0.788**	0.151	0.724
6	R	I intend to develop skills in accessing, acquiring and appraising evidence relevant to my area of practice	0.125	0.152	**0.779**	0.141	0.756
8	R	I intend to apply best available evidence findings to improve practice	0.131	0.168	**0.765**	0.151	0.659
10	R	Literature and research findings are useful in my day-to-day work	0.066	0.114	**0.760**	0.169	0.646
13	R	EBP improves the quality of my work	0.168	0.056	**0.736**	0.246	0.714
14	R	EBP helps me make decisions about clients in my work	0.188	0.090	**0.723**	0.199	0.675
5	R	I intend to develop knowledge about EBP	0.068	0.036	**0.676**	0.106	0.691
28	P	Read published research reports related to EBP	0.379	0.135	0.166	**0.804**	0.652
26	P	Integrated research evidence with your expertise	0.389	0.054	0.222	**0.797**	0.671
29	P	Informally shared and discussed literature/research findings with others in your workplace	0.403	0.131	0.169	**0.794**	0.663
25	P	Critically appraised any literature you have discovered to determine the methodological quality	0.380	0.079	0.119	**0.771**	0.620
30	P	Formally shared and discussed literature/research findings with others in your department/practice	0.332	0.030	0.159	**0.770**	0.599
23	P	Tracked down the relevant evidence once you have formulated the question	0.454	0.089	0.128	**0.757**	0.609
22	P	Formulated a clearly answerable question that defines the client or problem, the intervention and outcome(s) of interest (Using PICO principles)	0.410	0.021	0.100	**0.733**	0.689
24	P	Searched an electronic database	0.244	0.118	0.085	**0.611**	0.650
27	P	Considered your clients’ preferences when making clinical/professional decisions	0.206	0.143	0.205	**0.565**	0.386
**Eigenvalues**			10.610	5.994	5.124	2.907	
**Variance (%)**			26.525	14.984	12.809	7.267	
**Cumulative (%)**			26.525	41.509	54.319	61.586	

Principal component analysis and Oblique rotation; C, T, R & P represent the domains of confidence, terminology, relevance and practice respectively

#### Confirmatory factor analysis

**Fig. 2:**
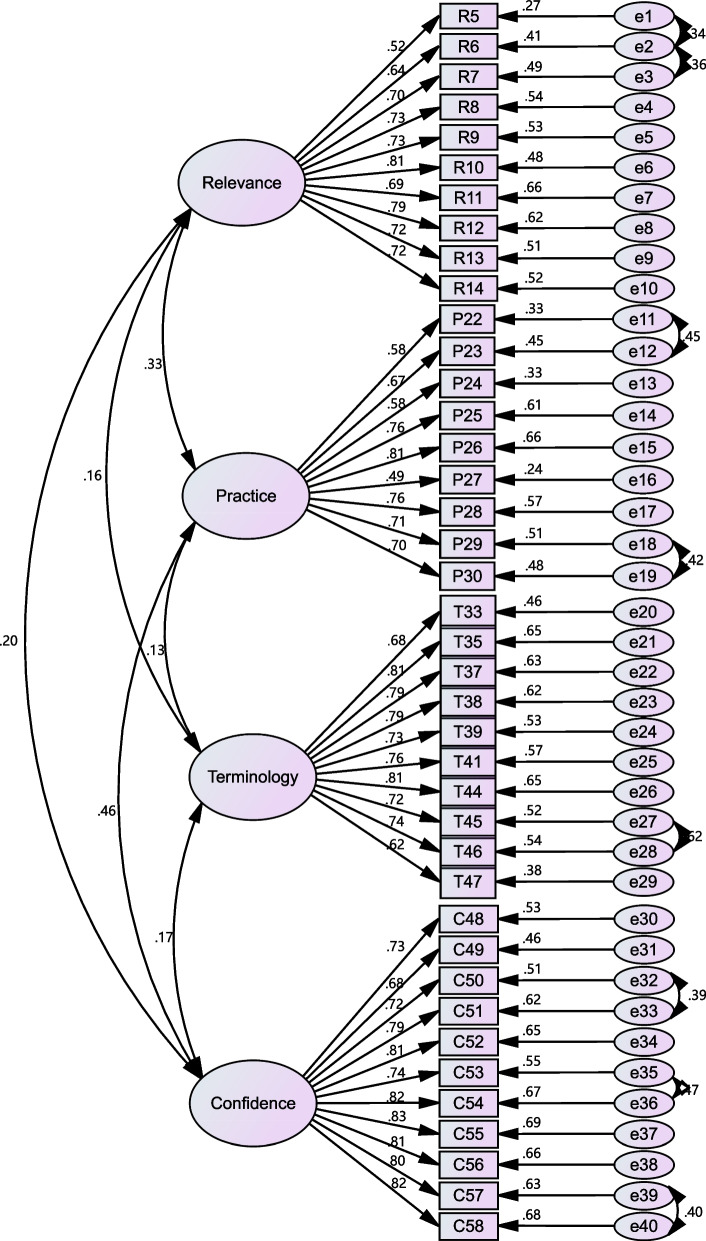
A schematic diagram of standardized model fitting of the Chinese EBP^2^Q (*n* = 320)

A total of 320 samples were employed for confirmatory factor analysis. A four-factor model was established according to the results of exploratory factor analysis (see Fig. 2 and Table 4). All fit indices within the initial model, except GFI and NFI, complied with suggested parameters for satisfactory model fitting. In the modified model, the fit indexes were excellent: the RMSEA was 0.052, less than 0.08; the GFI was 0.902, and NFI was 0.901 exceeding the benchmark of 0.90. Eventually, the four-factor model suitably fitted the survey data and its application was testified to be appropriate for the population surveyed.

**Table 4:** The fitting indexes of confirmatory factor analysis of the EBP^2^Q (*n* = 320)

Index	Benchmark	Initial Model	Modified Model
χ^2^/df	< 3	2.594	2.283
GFI	> 0.90	0.848	0.902
CFI	> 0.90	0.909	0.932
RMSEA	< 0.08	0.059	0.052
NFI	> 0.90	0.885	0.901
IFI	> 0.90	0.910	0.932
PCFI	> 0.50	0.847	0.860

#### Reliability

The summaries for internal consistency and split-half reliability of the EBP^2^Q are illustrated in Table 5. The Cronbach’s alpha for the overall questionnaire was 0.926 and the four domains had the Cronbach’s alpha of 0.921 (Relevance), 0.894 (Practice), 0.922 (Terminology) and 0.950 (Confidence). Split-half reliability of all items of Chinese version of the EBP^2^Q was 0.925 and values for the four domains ranged from 0.848 to 0.926. Test-retest reliability by ICC was 0.868 for the overall questionnaire and 0.719 to 0.805 for the four domains.

**Table 5:** Reliability analysis of the EBP^2^Q (*n* = 633)

Domains	No. of items	Cronbach’s α	Split-half	Test-retest
Relevance	10	0.921	0.852	0.738
Practice	9	0.894	0.848	0.805
Terminology	10	0.922	0.872	0.719
Confidence	11	0.950	0.926	0.779
EBP^2^Q	40	0.926	0.925	0.868

## Discussion

In this study, we cross-culturally adapted the EBP^2^Q in Mainland China, providing an effective tool for evaluating the EBP learning outcomes of clinical postgraduates, especially for the clinical professional degree postgraduates. Chinese version of the EBP^2^Q introduced in this study contains 40 items, including four domains: Relevance, Practice, Terminology and Confidence. Relevance domain (10 items) measures students’ attitude towards EBP. Practice domain (9 items) measures the frequency of applying EBP in clinical situations. Terminology domain (10 items) measures students’ understanding of EBP related terms. Confidence domain (11 items) measures students’ self-confidence in their EBP related skills.

The items in the Sympathy domain and several items in the Relevance domain and the Terminology domain were deleted according to the results of content validity and experts’ reviewing. On one hand, some items (items 17–19) of the Sympathy domain involved the accumulation of clinical work experience, which was not suitable for medical students to answer. On the other hand, the other items (items 15, 16, 20 and 21) of the Sympathy domain expressed similar meanings to the contents in the items of the Relevance domain. Some terms in the Terminology domain, such as “relative risk (RR)”, “absolute risk (AR)”, “number needed to treat (NNT)”, etc. were proper nouns in epidemiological studies. With reference to the results of CVI, these items were deleted to improve the generalizability of questionnaire. Meanwhile, the content validity indicated that the CVI of four items in the Relevance domain were low. The four items focused on the understanding of the concept of evidence-based practice, and were not related to the students’ attitude to the EBP, so these four items were also deleted.

The EBP^2^Q was developed and evaluated across a range of professions and showed acceptable psychometric properties. Maureen Patricia Mcevoy et al. [17] examined the questionnaire as a reliable instrument with the ability of monitoring changes in EBP learning outcomes after guidance or cumulative learning throughout the degree period for students or healthcare professionals with varying healthcare disciplines. Our project played a crucial role in the reliability and validity of the culturally adapted EBP^2^Q. The Cronbach’s alpha was 0.926 in 633 Chinese clinical postgraduates, indicating that good internal consistency was confirmed for the questionnaire. Similar value was also reported in the original version. The test-retest reliability of the adapted questionnaire was optimal in general and for each of the identified components. The ICC in 2-week retest indicated a strong reliability.

The CVI is the main method which is adopted to quantify content validity for multi-item instruments. S-CVI reached 0.938 in this study. I-CVIs were reported higher than 0.78 with eleven items in the Relevance and Terminology domain exception, and these items were removed at this stage. The results suggested the content validity of the EBP^2^Q was acceptable. Exploratory factor analysis indicated that the extracted four principal components accounted for 61.586% of the total variance, providing suitable indices for assessing the validity of this instrument. Our findings showed a strong similarity of factor structure with Polish version, indicating that the condition for theoretical validity is fulfilled. Polish version of the EBP^2^Q obtained five factors through exploratory factor analysis, which was consistent with the structure of the original version, while the item 15 from the Sympathy domain was included in the Relevance domain [32]. It confirmed the similar view in this study that some contents expressed in items of the Sympathy and Relevance domain were similar. Ming-Yu Hu et al. [19] conducted the exploratory factor analysis in a sample of Chinese clinical nurses, and obtained an eight-factor structure (the eight domains were redefined as basic understanding, intention, attitude, sympathy, clinical related terms, EBP related terms, practice and confidence according to their common characteristics.), which was inconsistent with our results. This may be resulted from the different characteristics between clinical postgraduates and nurses. The well-developed EBP curriculum system makes it accessible for clinical postgraduates to obtain EBP knowledge and skills, but less working experience limits the perception of the compatibility of EBP with clinical work.

As the further contribution of this study, confirmatory factor analysis (CFA) was conducted for investigating the fit of the four factors with the general structure of the EBP^2^Q. Model fit assessment plays a pivotal role in evaluating CFA models and the validity of psychological assessments. The fixed fit cutoffs utilized in the study are widely adopted in empirical research to identify potential model misspecification and select a concise model [33]. As suggested by Marsh HW [34], assessing goodness of fit is best achieved by considering multiple perspectives. It is typically recommended to examine several qualitative indices with well-established properties to evaluate model fit. Hence, Chinese version of EBP^2^Q was verified using 7 indices: χ^2^/df, GFI, CFI, RMSEA, NFI, IFI and PCFI. All the indices were satisfied the standard. The results of CFA indicated that the four-factor model with modification was considered a better fit, suggesting that the revised Chinese version of EBP^2^Q had good construct validity. Norwegian version of the EBP^2^Q [35] conducted confirmatory factor analysis to test whether the collecting data from 347 students majoring in social education and nursing fit the original five-factor structure. The results of CFA did not confirm the original five-factor model (CFI = 0.69, RMSEA = 0.09). In addition, there was no statistically significant difference in the domain of the Sympathy before and after the EBP course.

Allowing for the obtained results, Chinese version of EBP^2^Q consists of the four factors: Relevance, Practice, Terminology and Confidence. Each factor comprises a sufficient number of items. Moreover, the 40-item questionnaire has a high response rate and the pilot study has reassured a quality of the adapted version. Above all, the revised questionnaire has good reliability and validity, which can satisfy the domestic demand for relevant research and application.

## Limitation

Despite the result of the cross-cultural adaption of the EBP^2^Q is satisfied, several limitations should be mentioned. First, clinical postgraduates in our study were recruited using convenience sampling from three university affiliated hospitals in Northwest China, which may have impacted the widespread generalization and application of Chinese EBP^2^Q to some degree. However, the sample in this study has a broad range of specialties, clinical practice duration and research experience, suggesting that EBP^2^Q is understandable and acceptable by general Chinese clinical postgraduate’s population. Second, criterion validity or predictive validity was not directly determined because a gold standard does not exist. A psychometric assessment of EBP^2^Q with respect to convergent validity should be considered in the future validation study. Third, while the fixed fit cutoffs employed for model fit assessment in CFA have gained significant recognition, methodological research has highlighted that cutoff values may vary depending on the characteristics of the data and model being evaluated [36]. Daniel McNeish et al. proposed a simulation-based approach known as dynamic fit index cutoffs, which allows fit index cutoffs in CFA models to be dynamically adjusted to align with data and model characteristics. However, the widespread application and validation of this approach are still limited. Due to the nature of dynamic cutoffs, additional computations are required, making it more complex than fixed fit cutoffs and potentially leading to a higher likelihood of user errors [33]. Thus, we continued to utilize fixed fit cutoffs in this study. The possibility of dynamically adjusting fit index cutoffs based on specific model and data characteristics will be considered in future studies.

## Conclusion

Chinese version of the EBP^2^Q possesses adequate validity, test-retest reliability and internal consistency. The results indicate that the tool is replicable and applicable for EBP learning outcomes evaluation of clinical postgraduates. Chinese version of EBP^2^Q could be adopted by medical educators in designing their course and curriculum, or by clinical postgraduates for self-assessment of EBP learning outcomes.

## Data Availability

The datasets used and/or analyzed during the current study are available from the corresponding author on reasonable request.

## Abbreviations

EBP: Evidence-based practice
EBP^2^Q: Evidence-based practice profile questionnaire
ACE tool: Assessing competency in evidence based medicine
SPSS: Statistical package for the social sciences
SD: Standard deviations
CVI: Content validity index
S-CVI: Scale level of content validity index
I-CVI: Item level of content validity index
ICC: Intraclass correlation coefficient
PCA: Principal component analysis
EFA: Exploratory factor analysis
CFA: Confirmatory factor analysis
RMSEA: Root mean squared error of approximation
CFI: Comparative fit index
GFI: Goodness-of-fit index
NFI: Normed fit index
IFI: Incremental fit index
PCFI: Parsimony-adjusted comparative fit index
CR: Critical ratio
KMO: Kaiser-Meyer-Olkin
RR: Relative risk
AR: Absolute risk
NNT: Number needed to treat
